# Effect of food on the pharmacokinetics of the WEE1 inhibitor adavosertib (AZD1775) in patients with advanced solid tumors

**DOI:** 10.1007/s00280-020-04101-4

**Published:** 2020-06-16

**Authors:** Mats Någård, Mei-Lin Ah-See, Karen So, Marit Vermunt, Fiona Thistlethwaite, Mariette Labots, Patricia Roxburgh, Alain Ravaud, Mario Campone, Liselot Valkenburg-van Iersel, Lone Ottesen, Yan Li, Ganesh Mugundu

**Affiliations:** 1 Clinical Pharmacology and Quantitative Pharmacology, R&D Clinical Pharmacology and Safety Sciences, AstraZeneca, Gaithersburg, MD USA; 2 Late Stage Development, Oncology R&D, AstraZeneca, Cambridge, UK; 3 The Netherlands Cancer Institute-Antoni van Leeuwenhoek Hospital, Amsterdam, The Netherlands; 4 The Christie NHS Foundation Trust and University of Manchester, Manchester, UK; 5 Cancer Center Amsterdam, Amsterdam UMC, Amsterdam, The Netherlands; 6 Beaston West of Scotland Cancer Centre and University of Glasgow, Glasgow, UK; 7 Bordeaux University Hospital, Bordeaux, France; 8 ICO – Site René, Saint Herblain, France; 9 Maastricht University Medical Centre, Maastricht, The Netherlands; 10 Clinical Pharmacology Biologics and Bioanalysis, Clinical Pharmacology and Safety Science, BioPharmaceuticals R&D, AstraZeneca, Boston, MA USA; 11 Clinical Pharmacology and Quantitative Pharmacology, R&D Clinical Pharmacology and Safety Sciences, AstraZeneca, 35 Gatehouse Drive, Waltham, MA 02451, USA

**Keywords:** Adavosertib, Food effect, Pharmacokinetics, Oncology, WEE1

## Abstract

**Purpose:**

To support future dosing recommendations, the effect of food on the pharmacokinetics of adavosertib, a first-in-class, small-molecule reversible inhibitor of WEE1 kinase, was assessed in patients with advanced solid tumors.

**Methods:**

In this Phase I, open-label, randomized, two-period, two-sequence crossover study, the pharmacokinetics of a single 300 mg adavosertib dose were investigated in fed versus fasted states.

**Results:**

Compared with the fasted state, a high-fat, high-calorie meal (fed state) decreased adavosertib maximum plasma concentration (*C*_max_) by 16% and systemic exposure (area under the plasma concentration–time curve [AUC]) by 6%; AUC_0–*t*_ decreased by 7% and time to maximum plasma concentration was delayed by 1.97 h (*P *= 0.0009). The 90% confidence interval of the geometric least-squares mean treatment ratio for AUC and AUC_0–*t*_ was contained within the no-effect limits (0.8–1.25), while that of *C*_max_ crossed the lower bound of the no-effect limits. Adverse events (AEs) related to adavosertib treatment were reported by 20 (64.5%) of the 31 patients treated in this study. Grade ≥ 3 AEs were reported by four (12.9%) patients (one in the fed state, three in the fasted state); two of these AEs were considered treatment-related by the investigator. Three serious AEs were reported in three (9.7%) patients; these were not considered treatment-related. No patients discontinued because of treatment-related AEs, and no new safety signals were reported.

**Conclusion:**

A high-fat meal did not have a clinically relevant effect on the systemic exposure of adavosertib, suggesting that adavosertib can be administered without regard to meals.

**Electronic supplementary material:**

The online version of this article (10.1007/s00280-020-04101-4) contains supplementary material, which is available to authorized users.

## Introduction

Adavosertib (AZD1775) is an orally active, first-in-class, small-molecule reversible inhibitor of WEE1 kinase [1]. WEE1 is a protein tyrosine kinase that phosphorylates and inhibits cyclin-dependent kinase 1 (CDK1), which drives cells from the G2 phase into mitosis, and CDK2, which drives cells through the S phase of the cell cycle [1, 2]. In response to DNA damage, WEE1 inhibits both CDK1, to maintain the cell in an inactive state and prevent mitosis, and CDK2, to delay the replication process and allow time for DNA repair [3]. Through inhibition of WEE1, adavosertib prevents G2 cell cycle arrest, leading to premature mitosis, and enhances CDK2 activation in cells in the S phase; this results in uncontrolled DNA replication and replication stress [1, 4]. The majority of human cancers have abnormalities in the p53 G1/S checkpoint, making them more dependent on the S and G2 checkpoints [1, 5]. The S and G2 checkpoint abrogation induced by adavosertib could selectively enhance killing of p53-deficient tumor cells when administered in combination with other anticancer agents [6], while single-agent activity may be achieved in tumor cells by inducing high levels of replication stress and/or endogenous DNA damage, resulting in mitotic catastrophe. With this in mind, adavosertib is being developed for use as a chemosensitizing agent in combination with one or more cytotoxic agents, or with olaparib, or with durvalumab, and as monotherapy for the treatment of advanced solid tumors.

The mechanism of action of adavosertib was confirmed in a Phase I study, which provided the first direct evidence of a reduction in phosphorylated Tyr15-Cdk (a target of WEE1 kinase) levels in paired tumor biopsies [7]. This study also provided concurrent evidence for a DNA damage response (based on increased levels of phosphorylated histone H2AX) and, thus, demonstrated the single-agent activity of adavosertib [7]. Other Phase I studies have assessed the safety and tolerability of adavosertib as monotherapy [8, 9] and in combination with chemotherapy, olaparib, or durvalumab in patients with advanced or refractory solid tumors [10–13]. Multiple dosing schedules have been evaluated for adavosertib mono- and combination therapy to maximize clinical activity and minimize toxicity [8, 9, 14].

In addition to these Phase I studies, Phase II data have shown adavosertib to have promising antitumor activity in women with *TP53*-mutated ovarian cancer refractory or resistant to first-line platinum-based chemotherapy when administered in combination with carboplatin [15]. Preliminary results from an open-label, four-arm, Phase II study of adavosertib plus four different types of chemotherapy regimens in patients with platinum-resistant ovarian cancer also suggested that adavosertib plus carboplatin shows promise in terms of efficacy [16]. Furthermore, a randomized Phase II study, also in women with platinum-resistant ovarian cancer, has demonstrated that adavosertib and gemcitabine combination therapy improves treatment efficacy compared with gemcitabine alone [17].

The pharmacokinetic parameters of single-dose adavosertib are approximately linear and increase in a dose-proportional manner [10]. Adavosertib is absorbed at a modest rate, with a median time to peak plasma concentration (*t*_max_) of 3–4 h and terminal half-life of 9.0–12.3 h following single doses of 325–1300 mg in patients with advanced solid tumors [10]. In vitro data indicate that the major pathway of adavosertib metabolism in humans involves CYP3A4, although FMO3 and FMO5 may also be involved [10], with no significant metabolites representing > 10% of unchanged adavosertib in human plasma.

It is critical that the effect of food on drug exposure is investigated early in clinical development because food can alter systemic exposure of an orally administered drug and mitigate or exacerbate toxicities [18, 19]. FDA guidance stipulates that food effect studies should be conducted early in the drug development process (ideally Phase I) in oncology [18, 20]. Knowing whether food affects the bioavailability of a drug before the pivotal trial can help to optimize study design, increase patient comfort by avoiding unnecessary fasting, reduce cost and ultimately improve efficiency in oncology drug development [18, 19]. The data from several food effect studies for orally administered cancer drugs were the subject of a review published in 2018 [18]. Of the 16 drugs with available data, only two had completed food effect data collection before the start of the pivotal trial. Consequently, for four of the 14 drugs with a food effect study that was completed after the pivotal study, the food restrictions on the label were different to those imposed in the trial.

To support dosing recommendations in future clinical studies, the current study (NCT03315091) assessed the effect of a high-fat, high-calorie meal (representing the setting of maximum possible perturbation of oral bioavailability in the postprandial state) on the pharmacokinetics (PK) of a single daily oral dose of 300 mg adavosertib, which is the recommended Phase II dose when administered as monotherapy [9].

## Materials and methods

### Objectives

The primary objective of the study was to investigate the effect of food on the PK of a single oral dose of 300 mg adavosertib in patients with advanced solid tumors. The safety and tolerability of adavosertib were also assessed.

### Study design

**Fig. 1:**
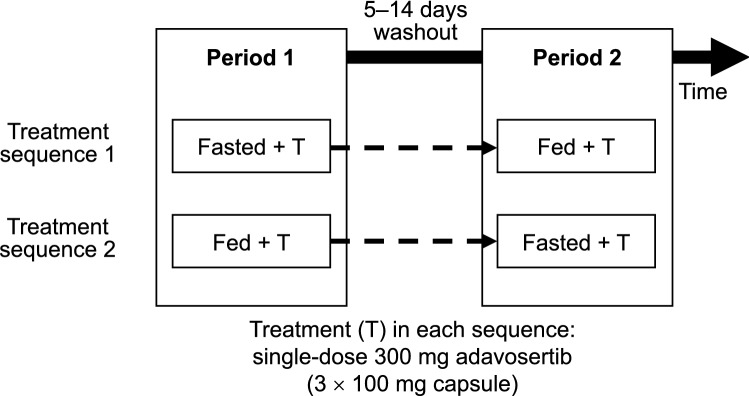
Study design. All test meals provided in the study were concordant with the recommendations of the FDA for a high-fat and high-calorie meal consisting of 50% calories from fat and 800–1000 calories in total [20]

This Phase I, open-label, randomized study had a two-period, two-sequence crossover design (Fig. 1) and was conducted in patients with advanced solid tumors for which standard therapy did not exist or had proven ineffective or intolerable.

Patients were screened within 28 days prior to day 1 of the first treatment period and randomly assigned to one of two treatment sequences before administration of the first dose of the study drug. In treatment sequence 1, patients received a single oral dose of 300 mg adavosertib after an overnight fast of at least 10 h and continued fasting for at least 4 h after administration (fasted state). After a washout period of at least 5 days, but no more than 14 days, patients received another single oral dose of 300 mg adavosertib within 30–45 min after having initiated consumption of a high-fat, high-calorie meal (fed state) [20]. In treatment sequence 2, patients received a single oral dose of 300 mg adavosertib first in the fed state, followed by the same washout period and another single oral dose of 300 mg adavosertib in the fasted state.

All test meals complied with FDA recommendations for a high-fat and high-calorie meal, where fat comprised approximately 50% of the total calorific content of the 800- to 1000-calorie meal [20]. An example test meal included 150 mL whole milk (3% fat), 45 g cereal (cornflakes), half a slice of fried bread, 60 g lean back bacon, one lightly fried egg, three slices of toast, 30 g butter, and 200 mL decaffeinated tea/coffee (with milk, which was part of the 150 mL allowance).

Patients stayed in the study center overnight to optimally manage fasting and food consumption, and all patients received intravenous anti-emetic medication (granisetron 1 mg or ondansetron 8 mg) within 30–40 min prior to adavosertib administration. In the fed state, patients were considered evaluable provided they consumed at least 75% of the high-fat meal within 45 min. If the patient did not consume at least 75% of the meal within 45 min, they were not dosed or sampled for PK in that treatment period. If a patient vomited within 3 h after adavosertib administration, all PK sampling ceased for that treatment period.

The study was performed in accordance with the Declaration of Helsinki, Good Clinical Practice, applicable regulatory requirements and the AstraZeneca policy on bioethics [21]. Following the end-of-treatment assessment, patients could enroll in a continued-access study (NCT03313557) to receive adavosertib at the recommended Phase II dose of 300 mg per day on days 1–5 and 8–12 of a 21-day treatment cycle.

### Patients

Both male and female patients of at least 18 years of age were eligible for inclusion in this study. Key inclusion criteria were as follows: patients had to have a histologically or cytologically documented locally advanced or metastatic solid tumor, excluding lymphoma, for which standard therapy did not exist or had proven ineffective or intolerable; an Eastern Cooperative Oncology Group (ECOG) performance status of 0 or 1; any prior palliative radiation had to have been completed at least 7 days before the start of study treatment, and patients had to have recovered from any acute adverse effects. Required thresholds for baseline laboratory values (within 7 days of study treatment initiation) were: absolute neutrophil count ≥ 1500/μL; hemoglobin ≥ 9 g/dL; platelets ≥ 100,000/μL; alanine aminotransferase and aspartate aminotransferase ≤ 3 × upper limit of normal (ULN), or ≤ 5 × ULN if the patient had documented hepatic metastases; serum bilirubin within normal limits, or ≤ 1.5 × ULN in patients with hepatic metastases; and serum creatinine ≤ 1.5 × ULN. Patients had to be able to consume a high-fat meal. Key exclusion criteria were as follows: known malignant central nervous system disease other than neurologically stable, treated brain metastases; use of any anticancer treatment drug ≤ 21 days or ≤ 5 half-lives (whichever was shorter) prior to first administration of the study drug; patients suffering from conditions that were likely to adversely affect gastrointestinal motility and/or transit (including diarrhea, vomiting and nausea), or with gastrointestinal resection likely to interfere with absorption of study treatment; and patients who were dependent on a medication that could adversely affect gastrointestinal motility or transit.

Patients were not permitted to use any other anticancer therapy, biologic therapy or novel investigational agent during the study treatment. All patients had to avoid concomitant use of medications, herbal supplements, and ingestion of foods with known inducer/inhibitory effects on CYP3A4. Loperamide was permitted during the study for treatment of diarrhea.

### Assessments

Pharmacokinetic samples and safety assessments were obtained pre-dose and up to 72 h post-dose (every 15 min up to 1 h post-dose, hourly from 1 to 4 h post-dose, bi-hourly from 4 to 12 h post-dose, every 12 h from 12 to 48 h post-dose, and at 72 h post-dose) in each treatment period. The concentration of adavosertib in human plasma, containing dipotassium ethylenediaminetetraacetic acid as an anticoagulant, was determined using protein precipitation extraction followed by analysis with high-performance liquid chromatography and tandem mass spectrometric detection. The sample analysis was performed by Covance Laboratories (Madison, WI, USA) using the bioanalytical method initially developed and validated by Merck (described previously [22]), which has been transferred and cross-validated by Covance Laboratories for sample analysis in clinical trials. All samples were stored at − 70 °C prior to analysis and analyzed within the validated time frame. A total of 939 samples were analyzed for this study. The adavosertib concentration in human plasma ranged from 4 to 2000 nM. The precision (coefficient of variation [CV]) was ≤ 4.7%, and the accuracy (percentage bias) of the quality control samples was within − 2.0% to − 0.4%. Statistical analysis was performed to determine the reliability of the analysis. Reproducibility was confirmed by re-analysis of 102 samples (with adavosertib concentrations above the lower limit of quantification) selected at random: 99.0% of the results obtained following the initial and repeat analysis were within 20.0% of the mean of the two values, thus meeting the acceptance criteria [23].

#### Pharmacokinetic assessments

The following PK variables were measured: area under the plasma concentration–time curve from time zero to the time of the last quantifiable concentration (AUC_0–*t*_), calculated using the linear up, log down trapezoidal rule; area under the plasma concentration–time curve from time zero to infinity (AUC), obtained by extrapolating AUC to infinity using the terminal elimination rate constant; and maximum plasma drug concentration (*C*_max_). To characterize the response, the following PK variables were measured: *t*_max_; elimination rate constant (*λ*_z_); terminal half-life (*t*_½_); apparent clearance (CL/*F*); and apparent volume of distribution (*V*_z_/*F*).

#### Safety and tolerability assessments

Adverse events (AEs) were defined by the Medical Dictionary for Regulatory Activities (version 20.1) and graded by Common Terminology Criteria for Adverse Events (CTCAE; version 4.03). AEs were assessed by physical examination, monitoring of vital signs (blood pressure, pulse rate and body temperature), and evaluation of laboratory parameters (clinical chemistry and hematology). Safety assessments were performed up to 72 h post-dose in each treatment period. Upon completion of the 72-h safety assessment in treatment period 2, patients entered a 4-day washout period (relative to the last dose of adavosertib) and were required to attend an end-of-treatment assessment within 3 days of the washout period. AEs were captured throughout the study, starting 28 days prior to day 1 of the first treatment period until the end-of-treatment assessment. For patients who enrolled in the continued-access study, the end-of-treatment assessment was carried out within 3 days after the 4-day washout period relative to the last dose of adavosertib in treatment period 2; for patients who did not enroll in the continued-access study, this was within 30 days (permitted minus 7-day window) after the last dose of adavosertib.

### Statistical analyses

#### Determination of sample size

The size of this study was based on careful clinical consideration of the need to gain adequate PK data on the effect of food on adavosertib while exposing as few patients as possible to study procedures. No formal hypothesis testing was planned. Interpretation of the results was based on the size of the treatment ratio and associated 90% confidence interval (CI). The intended enrollment was 24 patients in total, with the aim of ensuring 18 PK-evaluable patients; however, the study protocol allowed for enrollment of additional patients to ensure that there were at least 18 evaluable patients.

#### Analysis sets

The PK analysis set included all patients who received at least one dose of adavosertib in one treatment period and who had at least one quantifiable plasma concentration recorded post-dose without protocol deviations or events that would affect the PK analysis. The safety analysis set included all patients who received at least one dose of adavosertib.

#### Statistical methods

Statistical analysis was performed by IQVIA Early Clinical Development (ECD) under the direction of the AstraZeneca Biostatistics group and employed SAS^®^ version 9.4. Calculation of the PK parameters was the responsibility of IQVIA ECD Pharmacokinetics/Pharmacodynamics Department using non-compartmental methods with Phoenix^®^ WinNonlin^®^ version 6.4 (Certara, LP, Princeton, NJ, USA) and/or SAS version 9.4. For qualitative variables, the population size (N for sample size and n for available data) and the percentage (of available data) for each class of variable were reported. Quantitative variables were summarized using descriptive statistics, including n, arithmetic mean, standard deviation (SD), median, and minimum and maximum values. To assess the primary objective of this study, geometric mean ratios and 90% CIs of PK parameters were calculated based on a mixed-effects model, with fixed effects for sequence, period, and treatment, and subject nested within sequence as a random effect.

## Results

### Patients

A total of 37 patients were enrolled in seven study centers in France, the UK, and The Netherlands. There were six screening failures; therefore, 31 patients were randomized to one of the two treatment sequences. A total of 15 patients were assigned to treatment sequence 1 and 16 patients to treatment sequence 2. Three patients discontinued treatment because of worsening of the disease, resulting in 28 patients (14 per treatment sequence) who completed the study. As the three patients discontinued treatment after having received one dose of adavosertib (one in the fasted state and two in the fed state), data for the study safety and PK analyses were available for 29 and 30 patients in the fasted and fed states, respectively. Thus, data for all 31 randomized patients were included (Supplementary Fig. 1).

Fourteen patients had important protocol deviations. Of these, six were excluded from the PK analysis: one patient in the fasted state was excluded because it was uncertain whether movicolon had been withheld for 3 h before and after adavosertib dosing in accordance with the protocol; and five patients in the fed state were excluded, one because they received metoclopramide, potentially affecting the absorption of adavosertib, and four because they received adavosertib more than 45 min after the start of the meal. By accounting for these six excluded patients and the three patients who had discontinued because of worsening of the disease after the first treatment period, data were available for the final PK analysis for 28 and 25 patients in the fasted and fed states, respectively. A total of 22 patients had evaluable PK results for both the fed and fasted states.

Patient demographics and baseline characteristics were representative of the intended patient population and were generally balanced between treatment sequences (Table 1). A total of 19 (61.3%) of the 31 patients were female. The primary tumor location was varied; however, the most common primary tumor sites (in at least 10% of patients overall) were the ovaries in 6 (19.4%) patients and the rectum in 4 (12.9%) patients.

**Table 1:** Patient demographics and disease characteristics by treatment sequence

Characteristic	Fed–fasted	Fasted–fed	Total
	(*n* = 16)	(*n* = 15)	(*N* = 31)
Age, years			
Mean (SD)	60.6 (8.1)	62.5 (10.2)	61.5 (9.1)
Median (range)	59.5 (49–74)	64.0 (42–77)	61.0 (42–77)
Sex, *n* (%)			
Male	8 (50.0)	4 (26.7)	12 (38.7)
Female	8 (50.0)	11 (73.3)	19 (61.3)
Race, *n* (%)			
White	12 (75.0)	11 (73.3)	23 (74.2)
Missing	4 (25.0)	4 (26.7)	8 (25.8)
Body mass index, kg/m^2a^			
Mean (SD)	25.5 (2.9)	28.4 (8.9)	26.6 (5.9)
Median (range)	26.0 (20.1–28.6)	25.2 (20.5–45.0)	26.0 (20.1–45.0)
ECOG performance status, *n* (%)			
0: normal activity	7 (43.8)	5 (33.3)	12 (38.7)
1: restricted activity	9 (56.3)	10 (66.7)	19 (61.3)
Primary tumor location, *n* (%)			
Biliary tract	0	1 (6.7)	1 (3.2)
Breast	2 (12.5)	0	2 (6.5)
Cervix uteri	1 (6.3)	0	1 (3.2)
Colon	1 (6.3)	0	1 (3.2)
Esophagus	2 (12.5)	0	2 (6.5)
Head and neck	0	1 (6.7)	1 (3.2)
Kidney	0	2 (13.3)	2 (6.5)
Left parotid	1 (6.3)	0	1 (3.2)
Lung	2 (12.5)	0	2 (6.5)
Ovary	2 (12.5)	4 (26.7)	6 (19.4)
Pancreas	1 (6.3)	0	1 (3.2)
Penis	0	1 (6.7)	1 (3.2)
Rectum	2 (12.5)	2 (13.3)	4 (12.9)
Renal pelvis	0	1 (6.7)	1 (3.2)
Right parotid	0	1 (6.7)	1 (3.2)
Skin	0	1 (6.7)	1 (3.2)
Uterus	1 (6.3)	1 (6.7)	2 (6.5)
Uvea	1 (6.3)	0	1 (3.2)
Overall disease classification, *n* (%)			
Metastatic^b^	15 (93.8)	14 (93.3)	29 (93.5)
Locally advanced^c^	6 (37.5)	2 (13.3)	8 (25.8)
Previous chemotherapy, *n* (%)			
Yes	15 (93.8)	15 (100)	30 (96.8)
No	1 (6.3)	0	1 (3.2)
Number of regimens, *n* (%)			
1	2 (13.3)	2 (13.3)	4 (12.9)
2	3 (20.0)	2 (13.3)	5 (16.1)
3	1 (6.7)	5 (33.3)	6 (19.4)
4	4 (26.7)	1 (6.7)	5 (16.1)
5	2 (13.3)	2 (13.3)	4 (12.9)
6	1 (6.7)	2 (13.3)	3 (9.7)
7	0	1 (6.7)	1 (3.2)
8	2 (13.3)	0	2 (6.5)

A patient may have more than one disease classification, depending on the number of affected anatomical sites. ECOG performance status and overall disease classification are based on assessments at baseline. Primary tumor location is based on assessments at primary diagnosis

^a^Height and weight were determined for 16 and 30 patients, respectively. Body mass index was determined for nine patients in the fed–fasted and six patients in the fasted–fed treatment sequence, respectively

^b^Metastatic disease: patient has any metastatic site of disease

^c^Locally advanced: patient has any locally advanced site of disease

All patients participating in the study used concomitant medication (in addition to the mandatory pre-medication) during both treatment periods. Anti-emetic agents, including serotonin antagonists, were the most commonly used concomitant medication (used by 18 [62.1%] and 21 [70.0%] patients in the fasted and fed states, respectively), followed by pain medication, including opiates (used by 11 [37.9%] and 12 [40.0%] patients in the fasted and fed states, respectively) and non-steroidal anti-inflammatory drugs (5 [17.2%] and 6 [20.0%] patients in the fasted and fed states, respectively).

In accordance with the study protocol, all medications that could affect gastrointestinal motility or stomach acidity were prohibited to avoid alterations in the absorption of adavosertib. A total of 17 patients received one or more prohibited medications; of these, 12 patients were in both treatment groups. Most patients taking prohibited medications received laxatives for the treatment of constipation or other non-parenteral medications for the treatment of diarrhea or other gastrointestinal symptoms. However, the study centers dosed these medications at least 3 h before or after each adavosertib administration, so their use was not expected to affect adavosertib PK results.

### Pharmacokinetic assessments

The adavosertib PK parameters are summarized by treatment group (PK analysis set) in Table 2. The geometric mean AUC and AUC_0–*t*_ values were similar whether adavosertib was administered in the fasted or fed state. The geometric mean *C*_max_ was 19% lower in the fed versus fasted state (965.9 vs 1192 nM, respectively).

**Table 2:** Summary of adavosertib PK parameters by treatment group (PK analysis set)

Parameter	Fed (*n* = 25)			Fasted (*n* = 28)		
	Mean (SD)	Geometric mean (CV, %)	Median (range)	Mean (SD)	Geometric mean (CV, %)	Median (range)
AUC (nM h)	15,140 (5783)	14,080 (42.84)	14,410 (3620–33,600)	16,900 (4747)	16,300 (27.65)	17,370 (10,400–31,000)
AUC_0–*t*_ (nM h)	14,820 (5693)	13,750 (44.06)	13,980 (3390–32,600)	16,640 (4737)	16,040 (28.05)	17,020 (10,400–30,800)
*C*_max_ (nM)	1012 (301)	965.9 (33.00)	1010 (390–1630)	1244 (352.9)	1192 (31.56)	1260 (439–2120)
*t*_max_ (h)	ND	ND	5.95 (2.93–8.12)	ND	ND	3.01 (1.00–6.05)
*t*_½_ (h)	12.29 (4.07)	ND	11.67 (6.84–28.1)	12.28 (3.708)	ND	11.20 (7.54–25.7)
CL/*F* (L/h)	46.05 (26.85)	ND	40.85 (17.5–163)	37.40 (10.08)	ND	33.88 (19.0–56.5)
*V*_z_/*F* (L)	916.5 (1200)	ND	640.0 (338–6590)	670.3 (308.9)	ND	578.4 (303–1660)

*CV* coefficient of variation, *ND* not determined

**Fig. 2:**
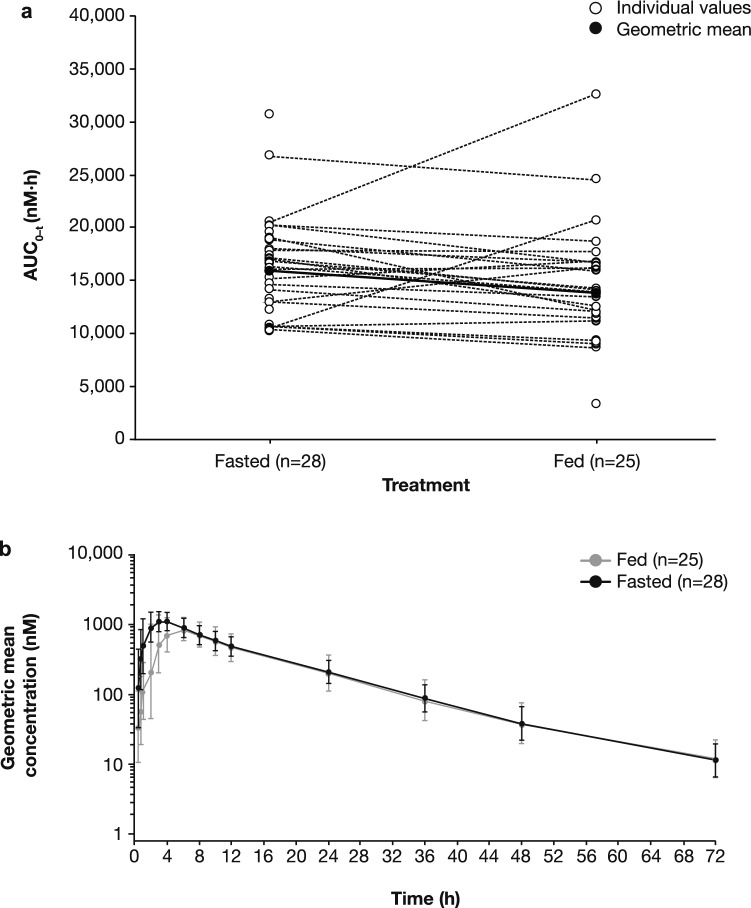
**a** Individual and geometric mean AUC_0–*t*_ of adavosertib by treatment group (PK analysis set). **b** Geometric mean (± geometric SD) plasma concentration of adavosertib by treatment group over time (PK analysis set)

Systemic exposure was lower in the fed state compared with the fasted state, as shown for AUC_0–*t*_ in Fig. 2a. Two patients showed pronounced higher systemic exposure in the fed state compared with the fasted state. Furthermore, the systemic exposure for one patient in the fed state was lower than for other patients under the same conditions (Fig. 2a). These outliers may account for the approximate tenfold difference in the median value ranges of AUC and AUC_0–*t*_ in the fed state compared with the approximate threefold difference in median value ranges seen in the fasted state reported in Table 2. No events or patient characteristics were identified that may explain these observations.

Geometric mean adavosertib plasma concentration over time is shown in Fig. 2b. Overall, the presence of food resulted in slower initial absorption in the fed state versus the fasted state, leading to lower initial plasma concentrations. By 6 h after administration of adavosertib, geometric mean concentration–time profiles were comparable between groups for the remaining time course. Geometric mean plasma concentrations peaked at 6 h in the fed state and 4 h in the fasted state.

Statistical comparisons of adavosertib primary exposure parameters (AUC, AUC_0–*t*_, and *C*_max_), as well as *t*_max_, are summarized in Table 3. Statistical analysis confirmed that systemic exposures to adavosertib for AUC, AUC_0–*t*_, and *C*_max_ were similar in the fed and fasted states. Co-administration of adavosertib with food decreased adavosertib geometric least-squares (LS) mean systemic exposure by 6% (AUC) and 7% (AUC_0–*t*_), and decreased geometric LS mean *C*_max_ by approximately 16%, compared with adavosertib administered in the fasted state. The 90% CIs of the geometric LS mean treatment ratios for AUC and AUC_0–*t*_ were fully contained within the no-effect limits of 0.8 and 1.25, while that of *C*_max_ crossed the lower bound of the no-effect limits. Administration of adavosertib in the fed state delayed median *t*_max_ by 1.97 h compared with the fasted state, which was statistically significant (*P *= 0.0009).

**Table 3:** Statistical comparison of key adavosertib PK parameters in the fed and fasted treatment groups

Parameter	Fed (*n* = 25)	Fasted (*n* = 28)	Fed/fasted ratio	
	Geometric LS mean		Ratio	90% CI
AUC (nM h)	14,610	15,620	0.9353	0.8541–1.0243
AUC_0–*t*_ (nM h)	14,260	15,370	0.9276	0.8442–1.0193
*C*_max_ (nM)	988.7	1172	0.8434	0.7538–0.9436

Parameter	Median		Fed/fasted comparison	
			Difference	90% CI
*t*_max_ (h)	5.95	3.01	1.97^a^	1.03–2.58

Results for AUC, AUC_0–*t*_ and *C*_max_ are based on the linear mixed-effects model, with sequence, period, and treatment as fixed effects and subject nested within sequence as a random effect. Treatment group medians for *t*_max_ are based on all patients with *t*_max_ estimates in the respective treatment group. Median differences and CIs were calculated using the Hodges–Lehmann estimator. *P* values for treatment group difference in median *t*_max_ were calculated using the Wilcoxon signed rank test

^a^*P *= 0.0009

Median (range) *λ*_z_ was 0.595 (0.0247–0.1013) for patients in the fed state and 0.0612 (0.0270–0.0919) for patients in the fasted state. Other PK parameters of interest are summarized by treatment group in Table 2. Median *t*_max_ was 5.95 h in the fed state and 3.01 h in the fasted state. Arithmetic mean *t*_½_ of adavosertib was similar whether administered in the fed or fasted state: 12.29 and 12.28 h, respectively. Arithmetic mean CL/F was 23% (46.05 vs 37.40 L/h) higher, and arithmetic mean *V*_z_/*F* was 37% (916.5 vs 670.3 L) higher, when adavosertib was administered in the fed versus fasted state, respectively.

### Safety

Adverse events by category are summarized in Table 4. At least one AE of any cause and grade occurred in 30 (96.8%) of the 31 patients in the safety analysis set. In the fed treatment group, 25 (83.3%) patients experienced AEs, while 23 (79.3%) patients experienced AEs in the fasted treatment group. One patient enrolled in the continued-access study 9 days after the end-of-treatment visit; for this patient, AEs were reported up to and including the date of enrollment in the continued-access study.

**Table 4:** Summary of AEs by treatment group (safety analysis set)

AE category	Number (%) of patients^a^		
	Fed (*n* = 30)^b^	Fasted (*n* = 29)^b^	Total (*N* = 31)
Any AE	25 (83.3)	23 (79.3)	30 (96.8)
Related to adavosertib treatment^c^	16 (53.3)	14 (48.3)	20 (64.5)
Any AE of CTCAE grade ≥ 3	1 (3.3)	3 (10.3)	4 (12.9)
Related to adavosertib treatment^c^	1 (3.3)	1 (3.4)	2 (6.5)
Any SAE	1 (3.3)	2 (6.9)	3 (9.7)
Related to adavosertib treatment^c^	0	0	0

AEs were defined according to Medical Dictionary for Regulatory Activities version 20.1

^a^Patients with multiple events in the same category are counted only once in that category. Patients with events in more than one category are counted once in each of those categories

^b^The columns ‘Fed’ and ‘Fasted’ represent all data from the fed and fasted periods irrespective of the treatment sequence the patient was assigned to

^c^As assessed by the investigator

The AEs that occurred most frequently (in at least two patients overall) are summarized in Supplementary Table 1 and included nausea and vomiting (both 38.7%), diarrhea and headache (both 19.4%), constipation (16.1%), and abdominal pain, anemia, cough, and fatigue (all 12.9%). Of the AEs that occurred in at least two patients, the following were more common in the fasted state than the fed state: vomiting (34.5% vs 20.0%); headache (20.7% vs 6.7%); constipation (17.2% vs 6.7%); abdominal pain (13.8% vs 0%); back pain (10.3% vs 3.3%); and pyrexia (10.3% vs 0%). AEs that were more common in the fed state than the fasted state included: nausea (33.3% vs 24.1%); diarrhea (16.7% vs 10.3%); and decreased appetite (10.0% vs 0%). There were no AEs that led to the discontinuation of adavosertib treatment.

AEs that were considered to be related to adavosertib treatment by the investigator were reported in 16 (53.3%) patients in the fed state and 14 (48.3%) patients in the fasted state (Supplementary Table 2). Three AEs considered by the investigator to be treatment-related were reported in at least two patients in both the fed and fasted states; these were nausea (38.7%), vomiting (35.5%), and diarrhea (16.1%). The following treatment-related AEs were more common in the fasted state than the fed state: vomiting (27.6% vs 20.0%), headache (10.3% vs 3.3%), fatigue (6.9% vs 3.3%), and abdominal pain (6.9% vs 0%). Treatment-related nausea (33.3% vs 24.1%) and diarrhea (13.3% vs 6.9%) were more common in the fed state than the fasted state.

No grade ≥ 4 AEs were reported. Four (12.9%) patients experienced a grade-3 AE: one patient in the fed state and three patients in the fasted state. In two of these cases, the AE was deemed to be treatment-related: diarrhea and headache in one patient in the fasted state, and hypokalemia in one patient in the fed state.

Three serious AEs (SAEs), which all led to hospitalization, were reported in 3 (9.7%) patients; none were considered treatment-related. Two patients experienced grade-1 SAEs, pyrexia and hydronephrosis, respectively, and one patient experienced a grade-3 SAE, urosepsis. There were no SAEs that led to the discontinuation of adavosertib treatment. No deaths were reported in the study.

No new clinically important findings or trends for safety laboratory parameters or vital signs were observed; however, some abnormal laboratory results were clinically significant and were reported as AEs. Notably, 1 (3.4%) patient in the fasted state (*n* = 29) experienced a single event of prolonged QTc (CTCAE grade 1), which was deemed treatment-related but was potentially confounded by hypomagnesemia (CTCAE grade 3). The event did not recur when the patient was dosed in the fed state.

## Discussion

The purpose of this Phase I, open-label, randomized, two-period, two-sequence crossover study was to assess the effect of food on the PK of a single oral dose of 300 mg adavosertib in patients with advanced solid tumors. Food is an important extrinsic factor that may influence the PK profile of a drug because it can change the extent of absorption, or delay absorption by decreasing the rate of gastric emptying [18].

The current study was designed to meet key FDA recommendations for a food effect study [18, 20]. Specifically, the FDA recommends a two-period, two-sequence crossover design testing a fed and a fasting condition to support the clinical development of a new agent. Furthermore, to generate the maximum possible effect on systemic drug availability, the fed group should receive a high-fat, high-calorie meal consisting of 50% calories from fat and 800–1000 calories [20]. The current study was not, however, conducted in healthy volunteers because adavosertib is a DNA damage response inhibitor, and it is neither safe nor ethical to administer this anticancer agent to healthy individuals. Therefore, the study was conducted in patients with advanced solid tumors for which standard therapy did not exist or had proven ineffective or intolerable.

The primary objective of this study was to investigate the effect of food on the PK of a single dose of adavosertib following oral dosing. Co-administration of adavosertib with food did not meaningfully affect its systemic exposure (AUC, AUC_0–*t*_, and *C*_max_) compared with administration in the fasted state. Co-administration of adavosertib with food decreased geometric LS mean systemic exposure by 6% (AUC) and 7% (AUC_0–*t*_), and decreased geometric LS mean *C*_max_ by approximately 16%, compared with the fasted state. The decrease in adavosertib exposure was not considered to be clinically relevant.

The secondary objective of this study was to characterize the PK of a single dose of adavosertib following oral administration. Adavosertib taken with food delayed the rate of absorption (median *t*_max_) by approximately 2 h compared with administration in the fasted state. The study also assessed the safety and tolerability of a single dose of adavosertib following oral administration. The safety profile of single-dose adavosertib in this study was consistent with its known safety profile as monotherapy [7, 8, 10]. The most prevalent AEs were gastrointestinal toxicities (nausea, vomiting and diarrhea). Four patients experienced AEs of grade 3 or worse; these were considered to be treatment-related in two patients (one in the fed state and one in the fasted state). Of the three SAEs reported in three patients, none were considered to be treatment-related. No significant AEs of clinical importance were observed in this study, nor were new safety signals identified.

The results of this study have several important clinical implications; for example, a lack of food effect minimizes the risk of unintended alterations to drug exposure, toxicity, and efficacy [24]. Furthermore, food restrictions associated with a dosing regimen can have a negative impact on patient adherence [25], especially when cancer patients are taking multiple agents with different and confusing instructions relating to dosing with food [19]. For cancer treatments that are subject to positive food effects, resources may subsequently be used in the development of alternative formulations that negate the restriction of a food label [26].

The study also has a number of limitations. Patients only received single doses of adavosertib; thus, although this is sufficient for PK assessments, no comparison could be made between the fed and fasted states in relation to toxicities occurring with the full treatment schedules, such as myelosuppression [7]. To that end, the AEs reported herein occurring in the fed and fasted states were observational; no formal analyses were performed, and it is not possible to conclude whether adavosertib is better tolerated when administered with or without food. The high-fat, high-calorie meal consumed by patients in this study was based on FDA guidance; therefore, the assumption was made that it would have the greatest effect on gastrointestinal physiology and would thus have the maximum effect on systemic drug availability. All patients in the present study received intravenous anti-emetic medication with granisetron or ondansetron prior to adavosertib administration; while no formal clinical drug interaction studies have been performed with adavosertib, a potential drug–drug interaction seems unlikely based on the low potential of these agents to significantly induce or inhibit CYP450 enzymes. Finally, the small sample size was chosen to balance the need to gain adequate PK data against exposing as few patients as possible to study procedures. No formal hypothesis testing was planned, and no conclusions can be drawn in relation to the efficacy of adavosertib.

In summary, administration of adavosertib 300 mg as a single oral dose following a high-fat, high-calorie meal did not have a clinically relevant effect on its systemic exposure, and the data from this study suggest that adavosertib can be administered in a fasted or fed state. Excluding SAEs and events leading to discontinuation of study treatment, no other significant AEs of clinical importance were observed in this study. Furthermore, no new safety signals were identified in this study.

## Electronic supplementary material

Below is the link to the electronic supplementary material.

Supplementary material 1 (DOCX 40 kb)
