# Supplementary material for: Effect of food on the pharmacokinetics of the WEE1 inhibitor adavosertib (AZD1775) in patients with advanced solid tumors
Source: Cancer Chemother Pharmacol. 2020 Jun 16;86(1):97–108. doi: 10.1007/s00280-020-04101-4 (PMC7338825; doi:10.1007/s00280-020-04101-4)
Supplement: Supplementary file 1 — Supplementary material 1 (DOCX 40 kb) [file 280_2020_4101_MOESM1_ESM.docx]

# Effect of food on the pharmacokinetics of the WEE1 inhibitor adavosertib (AZD1775) in patients with advanced solid tumors

# Supplementary data

Supplementary Table 1. AEs that occurred in two or more patients

| MedDRA preferred term | Number (%) of patients^a^ | | |
| --- | --- | --- | --- |
|  | Fed (n=30)^b^ | Fasted (n=29)^b^ | Total  (N=31) |
| Patients with any AE | 25 (83.3) | 23 (79.3) | 30 (96.8) |
| Nausea | 10 (33.3) | 7 (24.1) | 12 (38.7) |
| Vomiting | 6 (20.0) | 10 (34.5) | 12 (38.7) |
| Diarrhea | 5 (16.7) | 3 (10.3) | 6 (19.4) |
| Headache | 2 (6.7) | 6 (20.7) | 6 (19.4) |
| Constipation | 2 (6.7) | 5 (17.2) | 5 (16.1) |
| Abdominal pain | 0 | 4 (13.8) | 4 (12.9) |
| Anemia | 2 (6.7) | 2 (6.9) | 4 (12.9) |
| Cough | 2 (6.7) | 2 (6.9) | 4 (12.9) |
| Fatigue | 2 (6.7) | 2 (6.9) | 4 (12.9) |
| Back pain | 1 (3.3) | 3 (10.3) | 3 (9.7) |
| Decreased appetite | 3 (10.0) | 0 | 3 (9.7) |
| Pyrexia | 0 | 3 (10.3) | 3 (9.7) |
| Abdominal distension | 2 (6.7) | 0 | 2 (6.5) |
| Dyspepsia | 1 (3.3) | 1 (3.3) | 2 (6.5) |
| Hyperthermia | 1 (3.3) | 1 (3.3) | 2 (6.5) |
| Hypokalemia | 1 (3.3) | 1 (3.3) | 2 (6.5) |
| Myalgia | 1 (3.3) | 1 (3.3) | 2 (6.5) |
| Urinary tract infection | 2 (6.7) | 0 | 2 (6.5) |

^a^Sorted in decreasing frequency of preferred term. Each patient with a specific event was only counted once for each preferred term. Where patients had multiple episodes of the same event, the unique preferred term was only counted once for that patient; ^b^AEs were recorded in the fed or fasted states according to the time of onset and irrespective of the assigned treatment sequence. If an event resolved during the washout period and recurred during the second treatment period, it would be counted twice for that patient. Unresolved events that worsened during treatment period 2 were counted as a new AE. MedDRA, Medical Dictionary for Regulatory Activities

Supplementary Table 2. Treatment-related AEs that occurred in two or more patients overall

| MedDRA preferred term | Number (%) of patients^a^ | | |
| --- | --- | --- | --- |
|  | Fed (n=30)^b^ | Fasted (n=29)^b^ | Total (N=31) |
| Any event related to adavosertib treatment^c^ | 16 (53.3) | 14 (48.3) | 20 (64.5) |
| Nausea | 10 (33.3) | 7 (24.1) | 12 (38.7) |
| Vomiting | 6 (20.0) | 8 (27.6) | 11 (35.5) |
| Diarrhea | 4 (13.3) | 2 (6.9) | 5 (16.1) |
| Fatigue | 1 (3.3) | 2 (6.9) | 3 (9.7) |
| Headache | 1 (3.3) | 3 (10.3) | 3 (9.7) |
| Abdominal pain | 0 | 2 (6.9) | 2 (6.5) |
| Anemia | 1 (3.3) | 1 (3.4) | 2 (6.5) |
| Constipation | 1 (3.3) | 1 (3.4) | 2 (6.5) |

^a^Sorted in decreasing frequency of preferred term. Each patient with a specific event was only counted once for each preferred term. Where patients had multiple episodes of the same event, the unique preferred term was only counted once for that patient; ^b^AEs were recorded in the fed or fasted states according to the time of onset and irrespective of the assigned treatment sequence. If an event resolved during the washout period and recurred during the second treatment period, it would be counted twice for that patient. Unresolved events that worsened during treatment period 2 were counted as a new AE;
^c^As assessed by the investigator

Supplementary Figure 1. Consolidated Standards of Reporting Trials (CONSORT) diagram showing the number of patients enrolled, randomized and analyzed in
this study


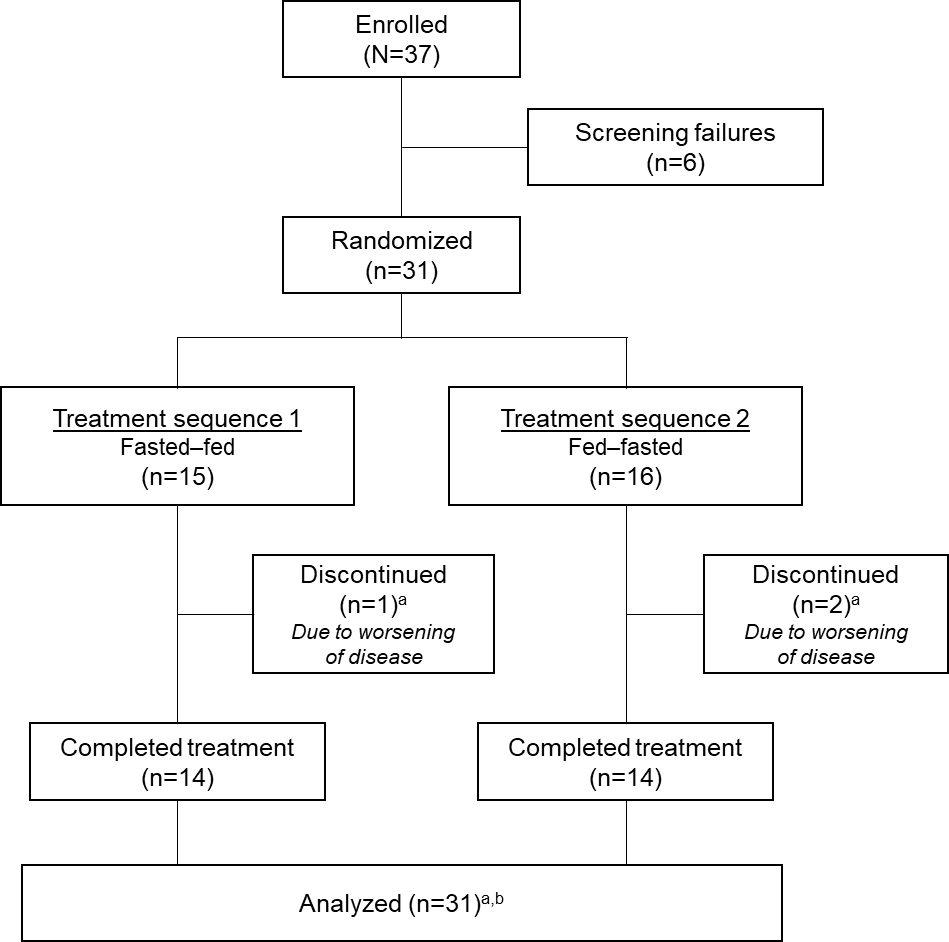


^a^All patients who received at least one dose of adavosertib were included in the analyses. For patients who discontinued after the first treatment (n=3), only data from that one treatment period were included in the analyses; ^b^As a result of the discontinuations, data were evaluable for 29 and 30 patients in the fasted and fed states, respectively
